# Social and Affective Neuroscience: Ensuring our future

**DOI:** 10.1093/scan/nsae035

**Published:** 2024-05-29

**Authors:** Matthew D Lieberman

**Affiliations:** University of California, Los Angeles, CA 90095-1563, USA

*Social Cognitive and Affective Neuroscience* is coming up on its 20th anniversary, and the contributing fields of social neuroscience and affective neuroscience are roughly 5 and 10 years older than that, respectively. Broadly speaking, social and affective neuroscience (SAN) has had a great run, far better than any of us there at the beginning had hoped for. Today, only one out of the top 30 psychology PhD programs in the U.S. (U.S. News & World Report, 2023), has no SAN faculty in their Psychology department and most of these departments have more than one of us.

Despite the clear success of our field, SAN is at a turning point where it can either accelerate its growth and gain its own institutional support system in perpetuity or embrace being ‘neither this nor that’ institutionally and depend on the continued interest of non-SAN faculty to extend our future. SAN could still ‘die on the vine’ if we fail to be strategic and proactive in the near future. Most top departments are already saturated with the 1–3 SAN faculty that operate as part of Social, Clinical, and Developmental areas of departments. Many other departments do not have the neuroimaging infrastructure or start-up funds to support the hiring of SAN faculty at all (but see the post-script if this applies to your department).

Given these limitations, how does SAN grow rather than stagnate over the coming decades? When I started out, there were only a handful of SAN labs in the U.S., and other departments eagerly snatched up our trainees, allowing SAN to grow quickly. Now there are more than 100 SAN labs in the U.S., each training quality PhD students, but there are not hundreds of SAN faculty positions waiting for them. So, what are we to do?

## We need SAN areas

I believe the answer lies in the active formation of SAN areas within Psychology departments. In departments that have formal areas, these areas are the primary leverage point of power in the department. Faculty commonly identify with their areas more than their departments and there is an inherent respect for areas. If a Social area has five faculty members and three leave or retire, the department will typically respond ‘We need to rebuild the Social area quickly’. But if a SAN faculty member leaves, few will say that this person *needs* to be replaced with another SAN researcher. Areas are self-sustaining in a way that the interests or methods of individual faculty members are not. When was the last time you saw an area disappear? This happens, but is not commonplace. But there are certainly niches within areas that departments move away from over time.

It is easy to take the area structure of our departments for granted. It seems like Social, Cognitive, Developmental, Clinical and Behavioral Neuroscience areas have been there forever because they were there before we were all undergraduates. In reality, departments did not always have these areas and small groups of people probably had to do hard work to convince their colleagues that the connected research interests of a group of faculty warranted its own area of the department. Social psychology has existed in some form since the late 1800s and was common in Psychology departments beginning in the 1930s and 40s. But prior to the 1950s and 1960s, there were almost no separate social psychology areas of departments. In each university, a handful of professors had to argue that a new area of the department would strengthen the department and keep it on the cutting edge. Today, there is nothing more mundane than a department having a Social area. SAN should be making the same argument today that social psychologists made in the 1950s, so that future generations will take for granted that every department needs a SAN area and that, of course, we need to make new SAN hires anytime a SAN faculty member leaves or retires.

At UCLA, four of us (Naomi Eisenberger, Carolyn Parkinson, Jaime Castrellon and myself) were able to get the first ever SAN area approved in our Psychology department this year. Frankly, it was harder than we thought it would be. It took 5 years of concerted effort to make this happen. We started planning in 2019 and first presented a proposal to our department’s executive committee in 2020. It got shot down. But they did offer us a path forward by supporting the portion of our plan that involved creating a SAN graduate major. We worked to get the SAN graduate major approved and have been taking new students into that major for the past 4 years. When we proposed the SAN area again in 2023, the fact that we had already administered a SAN major for a few years helped our case and we were approved for the new SAN area in early 2024.

If you have, say, three or more SAN faculty in your department, positioning yourself to become your own area has lots of upsides. But if your experience is anything like ours, you may encounter some resistance. I don’t think we were prepared for how much inertia and fear of change there is in Psychology departments. Perhaps SAN selects for people who have less of this fear, since by choosing SAN, you are still choosing a very young field that is not as established as other areas.

Below I describe some of the resistance points we encountered. Some may not stand in your way and obviously there might be others that we did not have to contend with. First, are the people who want to form a new area all coming from a single existing area that would be harmed by the loss of those people? This was a big issue for us. Three of us were coming from the Social area of our department and represented 40% of that area. The whole department was naturally concerned about the state this would leave the Social area in. We made a proposal that until both the SAN and Social areas strengthened, the teaching of the two areas should be considered together in terms of departmental expectations. We assured the department that there would be more of the classes the department cares about if were allowed to form a SAN area, than if we kept the status quo. Perhaps you will not run into this issue. Miraculously, the University of Delaware Social area organically transitioned from all non-neuroscience social psychology to all social neuroscience over the past decade and is in the process of changing their official area name to ‘Social neuroscience’. Second, faculty in the other areas of the department worried about whether a new SAN area would take resources from them and their areas. How you respond to this will depend on how your department allots these resources, but we were able to make a reasonably convincing case that this would not be a practical issue.

Apart from the effects on other areas, another big issue we faced was whether SAN *should* be an area. This argument came in two flavors. First, some argued that SAN at UCLA has already been successful while still being largely housed within the Social area. If it has been successful, why would we need to change what we have been doing. The second version of this criticism was along the lines of ‘SAN isn’t academically substantial enough to be its own area’. In other words, the argument was that SAN is a subarea of social psychology, the same way that memory research is a subarea of cognitive psychology. We don’t have any Memory areas of departments, so why should we have a SAN area?

## Is SAN a subarea of social psychology?

To respond to these concerns, we did what scientists do and looked at the data. Our conclusion was that SAN is an area, but that it ought to be compared more to newer areas like health psychology and cognitive neuroscience rather than longstanding areas in social, cognitive, and developmental psychology. I will have more to say about this below, but let’s start with the evidence that SAN is not a subarea of social psychology.

One of the nice things about creating a SAN area, rather than a social neuroscience area, is that it is easier to defend against the idea that it is a subarea of social psychology. While one could argue that social neuroscience was under the umbrella of social psychology at some point, affective neuroscience never was. Affective neuroscience’s history has more ties to behavioral neuroscience and clinical psychology with key early players like Richie Davidson, Jaak Panksepp, Richard Lane, Joe LeDoux and Liz Phelps.

Setting affective neuroscience aside for the moment, is social neuroscience even a subarea of social psychology? Whatever was true 20 years ago, the answer today is a clear ‘no’. Some of the best evidence for social neuroscience’s distance from social psychology comes from the ways that SAN researchers do and do not engage with social psychology. For instance, the flagship journal for social psychology is *Journal of Personality and Social Psychology* (JPSP). Journals in a field tend to be organized somewhat hierarchically, such that if you are a social psychologist you might publish in a more niche journal focused on a subarea (e.g. *Self & Identity*), but if you can, JPSP is where you try to publish since the piece will be seen by all social psychologists there. Since 2000, there have been 4690 articles published in JPSP. Of these, I could only find 7 focused on social neuroscience (i.e. ∼0.1%). Almost all of these were from before 2010. I obtained this number based on a variety of search terms like ‘MRI’, ‘EEG’, ‘neuroscience’ and so on. I’m sure I missed a few, but even still, it is clear that social neuroscientists almost never publish in JPSP. To my knowledge, SAN research is published even less frequently in other social psychology journals. If social neuroscientists were social psychologists they would publish in JPSP, but we are not so we do not.

**Fig. 1.**
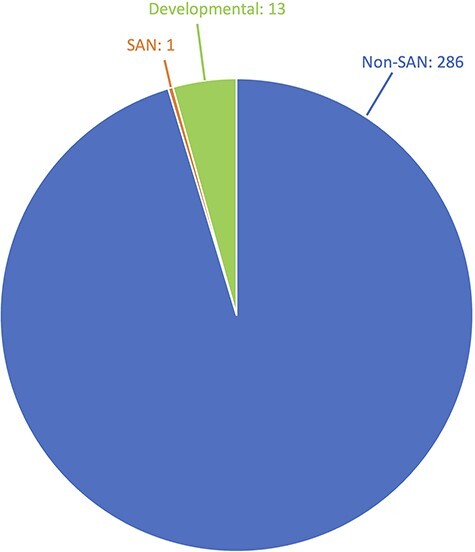
Distribution of SAN, developmental and other non-SAN talks given at the 2023 Society for Personality and Social Psychology Conference.

What about conferences? The *Society for Personality and Social Psychology* (SPSP) is the premiere social psychology conference. In 2023, there were at least 300 symposia talks given at SPSP. As shown in Figure 1, there was exactly one talk focused on SAN-related research. Contrast that with 13 developmental psychology talks given at SPSP. No one would confuse developmental psychology for being a subarea of social psychology, but it was far better represented at SPSP than social neuroscience. Again, SAN researchers largely do not see social psychology conferences as the place to present their SAN research.

**Fig. 2.**
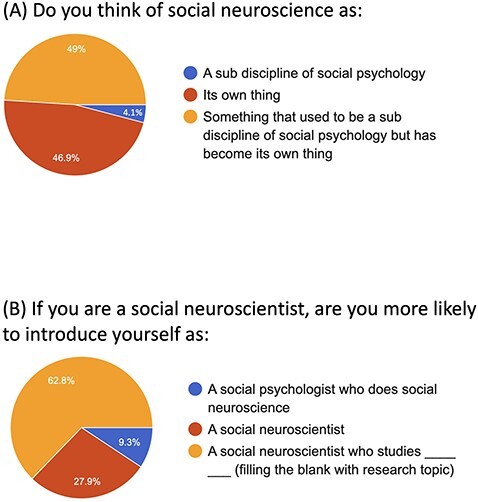
Among the membership of the SAN Society membership (a) the percentage of researchers who think of social neuroscience as a sub-discipline of social psychology and (b) the percentage who identify primarily as a social psychologist.

**Fig. 3.**
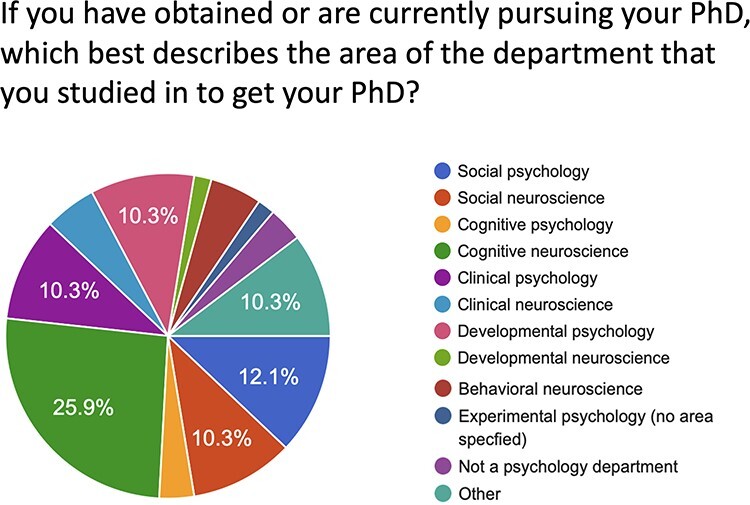
Percent of SAN PhD’s from different areas of psychology and neuroscience.

Finally, and most importantly, our UCLA SAN contingent conducted a survey of the membership of the *Social and Affective Neuroscience Society* using the society listserv and this provided invaluable data in demonstrating that SAN and social neuroscience are distinct from social psychology. We asked SAN researchers whether they thought of social neuroscience as: (i) a sub discipline of social psychology, (ii) its own thing, or (iii) something that used to be a sub discipline of social psychology but has become its own thing. As shown in Figure 2, only 4.1% indicated that they believe social neuroscience is a sub discipline of social psychology. Similarly, only 9.3% indicated that they would introduce themselves as ‘a social psychologist who does social neuroscience’ as opposed to a ‘social neuroscientist’ or ‘a social neuroscientist who studies ___’. This makes more sense in light of the fact that only 12% of SAN researchers are getting their PhD’s in social psychology (Figure 3). I think it would be valuable for the SAN society to commission a more systematic survey, as this would no doubt be helpful to future attempts to create SAN areas.

The preceding facts put SAN in a precarious situation. While other areas of your department might want to keep you in their area, rather than allowing you to defect and start a SAN area, this might be largely due to the teaching and service you provide to the area. This does not actually bode well for our future. Consider this: if you were a social psychologist and you were thinking about SAN researchers who do not publish in your journals, do not attend your conferences, and do not identify as social psychologists, would you want to replace existing SAN researchers with new SAN researchers in the future? If we look less like social psychology with each passing year, social psychology areas are likely to replace us with folks who *do identify* as social psychologists. This is a major reason why we need our own areas, our own power base, which will secure future faculty positions for our trainees.

## SAN is a standalone area

While the evidence is clear that SAN is not a satellite subarea in social psychology’s orbit, we still had to make the case that it rises to the level of a standalone area—more Mars than Pluto. The best argument is that we study a lot of questions that do not have non-neuroscience correlates in other areas. Even in just social neuroscience, we are much more than ‘social psychology in a scanner’. As time passes, we have developed our own inside baseball questions without reference to the long history of social psychological inquiry. Just to give a few examples (totally biased by my personal frame of reference): self–other differentiation, mirror neurons and action perception, autism, social working memory, neural activity at rest predicting subsequent social cognition, oxytocin and social bonds, the overlap of social and physical pain, domain-specific *vs* domain-general social perception, the relationship of social reward to financial reward, and the multi-sensory integration of faces and voices. In other words, we now have our own intellectual tradition. This was not always the case. When Kevin Ochsner and I wrote our early paper on social cognitive neuroscience (Ochsner and Lieberman, 2001), we *were* mostly focused on social psychology that could be done in the scanner—stereotyping, self-concept, and emotion regulation. Since then, social neuroscience has evolved and while each of these topics is still studied, they are not the core of our field.

There are other markers that anyone aspiring to create a SAN area can point to in order to demonstrate it warrants area-level status. We have multiple societies, conferences, and textbooks. The National Institutes of Health has a standing program on social and affective neuroscience analogous to its standing program on social and personality psychology. Similarly, when graduate students apply for a National Science Foundation fellowship, they list their field of study and ‘social/affective neuroscience’ is an option along with social psychology, cognitive psychology, developmental psychology and so on. There is no option for ‘memory research’ or ‘adolescent developmental psychology’. Thus, both of our major sources of funding already treat SAN as comparable to other ‘areas’ of typical psychology departments. One can now also point to the existence of a SAN area at UCLA and a soon-to-be Social neuroscience area at University of Delaware. If a few more SAN areas form, this will actually make it far easier for others to make the case that their department is being left behind by not forming a SAN area.

Another way to argue for the intellectual independence of SAN is to look at undergraduate teaching requirements. Every psychology department has courses they require for the undergraduate major, typically called ‘core’ or ‘distribution’ courses. These each represent a major independent area of psychological inquiry, with undergraduates required to take some combination of social psychology, developmental psychology, cognitive psychology, abnormal psychology and behavioral neuroscience often in the form of ‘choose one from list A and one from list B’. Currently, 8 of the top 30 psychology programs, according to *U.S. News & World Report*, have one or more SAN courses that count towards these core/distribution major requirements: Columbia, Princeton, NYU, University of Pennsylvania, Indiana University, University of Minnesota, UC Davis and UCLA.

You can also look to how many graduate applications your SAN-interested faculty are currently receiving relative to other areas of the department. We were able to do an analysis showing that at UCLA, the four faculty proposing to start a SAN area were receiving more applications than half of the current areas in our department. This suggests that from the standpoint of prospective graduate students, we were already operating at the level of an existing area.

## Prestige areas

One of the traps I fell into while initially arguing for a SAN area at UCLA, was trying to make the case that SAN looks just like every other legitimate area of the department. This was a losing argument because we do not. It was the wrong argument to make. SAN should not be compared with social psychology in 2024; it should be compared with social psychology in 1954 when it was just starting to break off from experimental psychology and become its own area of departments.

Given that the details of area formation in the 1950s are mostly lost to history, one could focus on comparisons to cognitive neuroscience and health psychology, which as disciplines are 10 and 20 years older than social neuroscience, respectively. The analogy of ‘social neuroscience is to social psychology as cognitive neuroscience is to cognitive psychology’ is quite clear and compelling. Both have historical roots in an area of psychology, but each has now gone on to distinguish itself intellectually, while still maintaining points of contact. Some at UCLA raised this as a point against us, as UCLA has a number of cognitive neuroscientists who are happily housed within the Cognitive area. However, if we look to the top 30 psychology programs in the U.S., eight have both a Cognitive area and a Cognitive neuroscience area (though not always with those exact names). In fact, five of the top ten have both, including both of the two most highly rated psychology departments.

Health psychology was another important comparison for us at UCLA, because we had already created a new Health area back in 2008. This was a departmental precedent that was easy to point to. On several metrics, SAN compares favorably. In 2008, there were almost no Health areas in other departments and no schools offered health psychology as a core course for the undergraduate major. In 2024, there are now a few SAN/social neuroscience/affective neuroscience department areas (UCLA and University of Delaware) and there are already at least eight schools offering SAN courses as a core course for the undergraduate major. Today, there are also almost 50% more SAN faculty than health psychology faculty at the top 30 psychology programs in the U.S. Indeed, while a third of these top programs have no health psychology faculty, only one has no SAN faculty. Additionally, of the top 30 programs, only UCLA, Carnegie Mellon, and Northwestern have Health areas today in 2024. Yet, at UCLA, we are thrilled that we have a Health area, because the field of health psychology is thriving and by having a Health area, we have announced to the world that we are one of the leaders in that field.

Beyond the traditional departmental areas, areas like cognitive neuroscience, health psychology and SAN are *prestige areas* that add a distinctive character to the department. They let the broader community know some of the specific ways a department is on the cutting edge of science. Other departments have prestige areas as well to indicate their unique strengths. UC Santa Barbara and UT Austin both have Evolutionary psychology areas because they have some of the foundational leaders of those areas. University of Pennsylvania has a Positive psychology area for the same reason. Similarly, University of Washington has an unusual concentration of clinical psychologists focusing on children, so they have a Child clinical psychology area in addition to Adult clinical psychology and Developmental areas.

**Fig. 4.**
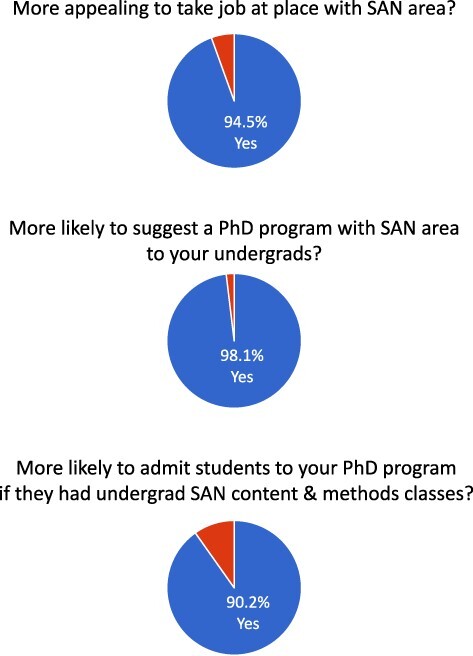
Survey of the Social and Affective Neuroscience Society membership regarding the prestige of a university with a formal Social and Affective Neuroscience area.

Each of these newer areas emerged from exciting trends in the field and distinguishes these departments from others in the field. Our survey of the membership of SANS suggests that the addition of a SAN area would produce a number of prestige effects for that department. Of the respondents, 95% indicated that a department with a SAN area would add to the appeal of being hired there as faculty (Figure 4). Similarly, 98% said they would be more likely to recommend a school with a SAN area to their undergraduates as they apply to graduate school. Finally, 90% indicated they would be more likely to admit an undergraduate to their PhD program if that student had both SAN content and methods classes as part of their undergraduate major, which is likely to occur in departments with formal SAN areas.

## Overcoming resistance

If your department supports you forming a new area, that is fantastic. Stop reading here, because you’re good. We definitely encountered some resistance in our department. Besides trying to encourage others to start SAN areas, the main purpose of this piece is to try to make it easier for you than it was for us. For instance, many of the statistics I’ve shared were part of the proposal that ultimately won over our department. Here are some things to consider that may help you make your case.

### Benefits to others

Some resources in a department are a zero-sum game. Our department is informally allowed to have a certain number of full-time faculty and in any given year there are a certain number of graduate student slots available for admitting new students. In light of these constraints, it is not surprising that our faculty were concerned that the creation of a new SAN area might divert resources that would otherwise go to other areas. If you can address this kind of issue directly in your own case, it is probably a good idea to do so.

Even if you cannot address it directly because their fears are warranted, you can potentially offset these concerns with other benefits that a SAN area will bring to the department. Besides arguing that SAN would bring prestige to UCLA, we focused on tangible benefits to other areas. For instance, by forming a new area, we would be offering new undergraduate courses that count towards the psychology major and this is a major need at UCLA. We also promised that we would prioritize the hiring of faculty who would teach graduate-level neuroimaging methods and analysis courses, which is a benefit to all of the non-SAN neuroimaging labs in the department. Finally, we focused on some area-specific benefits. With a SAN area, we would be hiring new affective neuroscientists which would provide training that the Clinical area is interested in. Furthermore, the kind of affective neuroscientist we can hire into a SAN area is different than those who might be hired into the Social area. If someone is hired into the Social area, they need to be able to teach part of the Social area curriculum. Freed from that burden, we are more likely to hire someone who works with animal models and would be more likely to collaborate with our Behavioral neuroscience area faculty.

### Find allies

If you do the above well, this will help you find allies who are not SAN faculty but will help promote your cause. One of the biggest mistakes I’ve seen faculty make is that they bring a proposal to the department, assuming they will win people over there, at the meeting. If you do things the right way, the faculty meeting is merely ceremonial. Make sure you have your support and the votes lined up *before* the meeting happens.

**Fig. 5.**
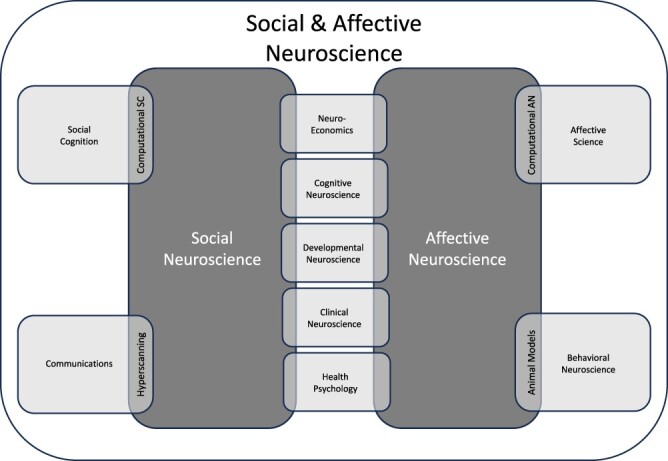
Allied research areas for Social and Affective Neuroscience.

One of the benefits of setting up a SAN area is that we have direct connections to so many areas of psychology and neuroscience. As you can see in Figure 5, there are people who do SAN research or care a lot about SAN research in most other areas of the department. At SCAN, I have consistently had associate editors who primarily identify with one of the other areas shown in Figure 5. Enlist these folks to help argue your case for you.

### Departmental history

Sometimes knowing the ancient history of your department can be helpful. If you don’t know it, there is probably someone who considers themselves the amateur historian of record for your department. Has an area formed in your department in the last 20 years? If so, how did that process go? And how does your department feel about that now? Are they glad they formed that area? Those can all be useful bits of information. Academics like precedent and if you can point to it and say ‘our department has done this before and it went well’, some resistance will fade. If your last new area was formed in a different millennium, you will really need to find that amateur historian.

### Join with others?

One question that came up multiple times for us was ‘Why don’t you join with the cognitive neuroscientists in the department and form a SCAN area or just call it cog neuro?’ There are a few ways to answer this question. For me personally, social and affective neuroscientists often care about questions that cognitive neuroscientists are indifferent too. And cleverness in SAN research often looks pretty different than cleverness in cognitive neuroscience research. I might be in the minority, but I don’t think SAN and cognitive neuroscience mesh particularly well. But that is not the issue here. I would have done almost anything to create a new area and would have been fine with SAN or SCAN or SAND. The bigger issue is that joining with others might produce unintended ripples within the department.

At UCLA, we have several cognitive neuroscientists within the Cognitive area who, as far as I can tell, are quite happy being in the Cognitive area. Similarly, my sense is that our cognitive psychologists really value having the cognitive neuroscientists in the Cognitive area. When our proposal went to the department, the Cognitive area faculty were broadly supportive of our proposal. Had we tried to peel off the cognitive neuroscientists to join us, it isn’t hard to imagine the same cognitive psychologists having a far less rosy view of our proposal. Obviously, these coalition politics will vary from department to department, but just think through the blowback that can occur with any moves you make.

### On the merits

Ultimately, you will need to make the case on the merits. This means making the case, as I did above, that SAN is really its own intellectual area with its own fertile questions that are not merely derivative of those from another area (i.e. social psychology in a scanner). This also means talking about the ways in which not having a SAN area is preventing faculty and students from having the best training and research environment in the program. Argue that you need a wider array of SAN faculty to really prepare students for the variety of subareas of SAN that they need to be able to train in.

Here’s another good argument that one of my colleagues came up with. As part of our proposal, we planned to set up a SAN talk series. A natural rejoinder to this was that the Social area talk series already has SAN speakers in it. This is true. However, these speakers would often give very different talks if they knew they were speaking with a dedicated SAN group rather than a Social area with lots of folks who do not use neuroimaging techniques. In the latter case, speakers tend to simplify their neuroimaging studies similar to when we speak to non-academic audiences. Even more problematic, the venue may well determine who is invited to speak. There are important ‘inside baseball’ talks about the latest advances in computational social or affective neuroscience that we would love to have in a SAN talk series that we would never invite as part of a Social area talk series. This is one element of a very practical case as to why SAN would provide better training for its students as its own area.

## Last word

This editorial is probably the last thing I will write for SCAN as Editor-in-Chief. We have seen so much growth in the field over the two decades that I have served as Editor. I never could have imagined it in my wildest dreams when Kevin Ochsner and I first started using the phrase ‘social cognitive neuroscience’ back in 1996. But we are at an inflection point, where we can either dig in and do the hard work to become one of the enduring areas of psychology that will still be making hires decades from now or we can leave SAN’s fate up to the whims of non-SAN researchers deciding whether to replace their current SAN faculty with new SAN faculty or instead move on to whatever is trendy in the future. From what I have observed, creating SAN areas in as many departments as possible over the next 20 years is the single most critical ingredient to protecting our field for our academic children and grandchildren. If I was the President of SANS, my entire mission would be to provide resources and support to SAN faculty to start SAN areas in their own departments. I certainly hope future Presidents of SANS will make it their mission too.

## P.S. Your department doesn’t have areas?

Everything I’ve written in this piece has been an argument for why the longevity of SAN as a field might depend on departments forming SAN areas and some of the considerations if you trying to move forward with doing that. Of course, this overlooks the fact that many departments do not have formal areas at all. I am certainly not proposing that if your department has no areas at all, that SAN should be the first. But I would suggest that if you are in a ‘no-areas’ department, start teaching large SAN lecture courses. Once you teach them, look into getting them listed as satisfying the core major requirements for undergraduate psychology majors. If more schools had SAN core requirements for the undergarduate major, it would make it easier for other departments with areas to argue that they should have a dedicated SAN area. SANS should keep a running list of schools that have SAN as part of the core requirements for the undergraduate major.

## P.P.S. Your department doesn’t support neuroimaging?

I am probably speaking to myself at this point, but just in case someone is reading this who is a SAN fan and would love to see SAN in their department even though their campus doesn’t support neuroimaging, this is for you. I recommend looking into hiring someone who does SAN functional near infrared spectroscopy (fNIRS) research. The start-up costs for hiring an fNIRS researcher are far lower than hiring an fMRI researcher. Furthermore, the only real costs are the equipment. I’d recommend buying two 16 × 16 NIRSport rigs which allows you to do Hyperscanning (or to scan one person at a time with whole head coverage). This equipment could also be purchased as an educational investment so the new professor can use the equipment when teaching neuroimaging methods, allowing your undergraduates to get hands on neuroimaging training that will improve their odds of getting into graduate school. While fNIRS has clear downsides compared with fMRI such as poorer spatial resolution and shallow cortical penetration, it has many clear upsides to a department without any human neuroimaging and to social neuroscience, specifically.

Besides the lower cost compared to fMRI, it is far more inviting to non-neuroscience students who would like to dip their toe in the neuroscience waters without committing their entire graduate career to it. fMRI has a 1–2 year learning curve, whereas fNIRS data collection can be mastered in a few days and the basics of fNIRS analysis can be learned in a month or two. Additionally, fNIRS allows for far more naturalistic research; fNIRS data can be collected during a hike in the woods. And the equipment is easy to take in carry-on luggage to another country for cross-cultural research (Burns *et al*., 2019; Dieffenbach *et al*., 2021). Finally, the capacity for hyperscanning opens up many potential research collaborations. You can look at teacher–student, parent–child and therapist–client interactions *in vivo*, as they are happening naturally. This invites collaborations with education, developmental and clinical researchers, respectively. If you only have a single fNIRS rig, you can run hyperscanning studies over Zoom with another lab that also has a rig (Binnquist *et al*.,). I suspect fMRI will be the ‘big dog’ in SAN for many years to come, but part of our growth as a field will need to come from schools that currently are not hiring SAN faculty at all because they think they cannot support the work financially. fNIRS and other more affordable brain-focused technologies (EEG, tDCS) may allow more universities to hire more SAN faculty and to make social neuroscience more social.
